# Using Visual Aesthetic Sensitivity Measures in Museum Studies

**DOI:** 10.3389/fpsyg.2020.00414

**Published:** 2020-03-10

**Authors:** Nils Myszkowski, Franck Zenasni

**Affiliations:** ^1^Department of Psychology, Pace University, New York City, NY, United States; ^2^Université de Paris, LAPEA, Boulogne-Billancourt, France; ^3^LAPEA, Univ. Gustave Eiffel, IFSTTAR, Versailles, France

**Keywords:** aesthetic abilities, aesthetic sensitivity, good taste, museum experience, psychological measurement

For over a century, differential psychologists (e.g., Cattell, 1890; Eysenck, 1940), educational psychologists (e.g., Thorndike, 1916; Seashore, 1929) and art theorists (e.g., Graves, 1948; Götz, 1985) have attempted to capture one's ability to form judgments of aesthetic objects that agree with external standards defined by stimulus construction criteria, layperson consensus, and/or expert consensus. In the visual domain, this ability—generally discussed as visual aesthetic sensitivity (Child, 1964) and measured through (notably) the Visual Aesthetic Sensitivity Test (VAST; Götz, 1985), its revision (VAST-R; Myszkowski and Storme, 2017), the Meier Art Tests (MAT; Meier, 1928) and the Design Judgment Test (DJT; Graves, 1948)—has recently regained interest, but has been mainly studied through its relations with individual differences in art expertise, personality, and intelligence among adults (e.g., Furnham and Chamorro-Premuzic, 2004; Myszkowski et al., 2014), and has remained unstudied in museum settings. In this paper, we review the current state of research on the validity of visual aesthetic sensitivity tests, and propose how to best implement them in museum studies.

## Elements of Validity of Visual Aesthetic Sensitivity Measures

Most frequently, visual aesthetic sensitivity tests operationalize Child's (1964) definition using “controlled alteration” (Meier, 1928, p. 188), a procedure which consists of deteriorating or creating an altered version of an aesthetic stimulus, and in presenting examinees with the altered and original stimuli, with the task of recognizing which is of better aesthetic quality. The construct validity of tests based on it are however controversial (Gear, 1986; Liu, 1990; Corradi et al., 2019), as it was notably argued that absolute aesthetic standards cannot exist, dismissing any operationalization of Child's definition. Nevertheless, the availability of *absolute* standards is not a necessary condition for the operationalization of Child's definition (Myszkowski et al., 2020): Aesthetic sensitivity tests rely instead on *empirical* standards, obtained through expert and/or laypeople consensus. Consequently, they compare an examinees' response with the typical response of experts—as originally suggested by Thorndike (1916)—or use expert agreement to select items—as used in the VAST. While using expert and/or laypeople consensus in lieu of absolute standards seems crude, it is actually common practice whenever *correctness* is not self-evident: It is for example used in the measurement of emotional intelligence (Mayer et al., 2003) or creativity (Amabile, 1982).

Still, using empirical standards poses the question of measurement (in)variance, especially across cultural backgrounds: Two artworks A and B may be aesthetically ordered as A > B for a group but as B < A for another. Fortunately, on that matter, studies of cultural measurement invariance—especially on the VAST (Iwawaki et al., 1979; Chan et al., 1980; Eysenck et al., 1984)—have provided encouraging results, with positive strong correlations between the item difficulties of the test across different groups differing in gender, age, and nationality (England, Japan, Hong Kong, Germany, and Singapore). More robust analyses (e.g., using differential item functioning), are certainly called for, but there is currently no empirical evidence of problematic measurement variance across cultures. We could speculate that the reason for this is that the controlled alteration method leads to examinees having to judge stimuli that are in the same (sub)category. Indeed, in visual aesthetic sensitivity tests, examinees do not compare Picasso's *Guernica* with Da Vinci's *Mona Lisa*—rather, they are asked to compare an original work of art with an almost identical (yet altered) version. Therefore, responding is less a matter of personal/cultural inclination regarding movements and styles, but more a matter of detecting an “out-of-tune” execution. It thus engages more the “ability to perform a set of basic perceptual analyses of the stimulus” (Myszkowski et al., 2014, p. 16) than one's ability to apply culturally relative norms.

Another sign of construct validity can be found in the concurrent validity of visual aesthetic sensitivity tests. This point is also quite controversial (Corradi et al., 2019; Myszkowski et al., 2020), but this is mainly because the nomological network of visual aesthetic sensitivity is yet to be clearly defined. Notably, Eysenck introduced confusion by originally discussing the construct as intelligence in the aesthetic domain (1940) to then speculate that the construct should be independent from intelligence (Frois and Eysenck, 1995)—which is contradicted in a recent meta-analysis (Myszkowski et al., 2018), which showed across 23 studies that its correlation with intelligence is significant and around 0.30. Nevertheless, one can reasonably expect that, as is found empirically, visual aesthetic sensitivity would be positively correlated with intelligence—because common cognitive processes are likely engaged in both measures (Myszkowski et al., 2018), and because it is common to observe relations between sensory perception in other domains and intelligence (e.g., Troche and Rammsayer, 2009)—or with personality traits like openness to aesthetics (Myszkowski et al., 2014)—because individuals with stronger interest in aesthetics may engage in more extensive processing, leading to higher accuracy, as it was for example found (Myszkowski, 2019) that, in these tests, response speed is negatively correlated with accuracy. Therefore, even though the nomological network of visual aesthetic sensitivity is not sufficiently (nor consistently) discussed, the pattern of relations between aesthetic sensitivity and other measures does suggest that visual aesthetic sensitivity measures present evidence of concurrent validity (Myszkowski et al., 2020).

These signs of validity could lead to a wide use of visual aesthetic sensitivity tests in the field where they would seem to belong: In contexts that naturally involve aesthetic judgments, such as museum visits. As they are however absent from museum studies, we will now discuss ways to facilitate their implementation in such contexts.

## How to Measure Visual Aesthetic Sensitivity in Museum Contexts

Because several visual aesthetic sensitivity tests are still in use, a first challenge could be to select one. Although these tests have showed satisfactory internal consistency in recent studies—with satisfactory Cronbach's αs (Furnham and Chamorro-Premuzic, 2004; Myszkowski et al., 2014; Summerfeldt et al., 2015)—their unidimensionality—a condition to even investigate internal consistency—and thus also their structural validity are largely unstudied. An exception is the VAST-R, which has been showed to present unidimensionality and structural validity—with a satisfactory fit of unidimensional Item-Response Theory models (Myszkowski and Storme, 2017). In addition, the VAST (and VAST-R) items present better evidence of content validity with the selection of the correct items by unanimity of a panel of 8 art experts (Götz et al., 1979). Finally, evidence of measurement invariance (though limited) is only provided for the VAST(-R) items (as discussed previously). Therefore, based on the current state of research we would suggest to prefer the VAST-R to other tests.

A second issue relates to scoring. While it seems straightforward to use sum/average scoring here, since the items of such tests are pass-fail items and vary greatly in difficulty (Myszkowski and Storme, 2017), one would advise to instead use Item-Response Theory (IRT) scoring. Using IRT in scoring such tests presents several advantages, such as obtaining conditional standard errors, which allows to identify cases that have been unreliably measured, or accounting for the guessing phenomena present in these tests. Still, using IRT remains challenging: It often requires specific training absent from many curricula (Borsboom, 2006) and demands large sample sizes for accurate estimation, which are not easily found in museum studies. Hopefully, regarding the VAST-R (other tests have not yet been studied with IRT), correlations between person estimates from (well-fitting) IRT models and sum/average scores are near perfect (Myszkowski and Storme, 2017). Therefore, even though IRT scoring is preferable, should IRT modeling not be possible, one could still use sum or average scores as an excellent proxy for IRT factor scores.

Related to technological advances, although this point remains unstudied, there is no evidence that these tests perform any differently when taken on-screen vs. in paper-and-pencil form: Both have been used indifferently. While measurement invariance between administration modalities needs empirical investigations, we could speculate that the two are equivalent. Actually, it may be more convenient in museum or virtual museum contexts to use tablets or computers for administration—smartphone screens are likely too small for properly displaying stimuli—and as we later suggest, there are psychometric advantages to using on-screen testing.

The use of computerized assessment first presents the practical advantage of allowing to reduce test length without compromising reliability, which would be desirable in assessing museum visitors. Because IRT models fit the VAST-R well (Myszkowski and Storme, 2017), researchers could use a Computerized Adaptive Testing (CAT) modified VAST-R, in which examinees would only take a subset of items that matches to their ability—re-estimated after each item—stopping assessment when such ability is estimated reliably enough (Green et al., 1984). The use of CAT is now largely facilitated by the availability of more software packages (e.g., Chalmers, 2016), and future studies may examine its usability with aesthetic sensitivity tests.

Further, as response times can be routinely collected when using computerized tests, we may suggest that recent IRT modeling advances in joint response and response time modeling could also allow to use response times as collateral information in the estimation of one's ability. Indeed, recent research (Myszkowski, 2019) suggests that there are strong dependencies between responses and response times (both related to a persons' speed and ability and to an item's difficulty and time intensity), which suggests that response times may be used to, for example, improve the accuracy of one's ability score, especially when fewer items are used (van der Linden et al., 2010). This could allow for even shorter tests, along with the improved detection of aberrant response/response times patterns (Marianti et al., 2014). As accuracy and speed are negatively correlated in the VAST-R, it has been also suggested (Myszkowski, 2019) to consider computing visual aesthetic sensitivity scores (accuracy scores) that are statistically controlled for response speed. This point is especially relevant for museum studies, because it is probably more likely to collect rushed responses from museum visitors than in experimental settings.

Finally, although we proposed that the VAST-R is the test that should currently be preferred, its content—black and white formal abstract paintings by Karl Otto Götz—remains rather narrow, and one may question the generalizability of the results of the test to other art styles and movements. We thus suggest that *ad-hoc* tests be built on a case-by-case basis using the controlled alteration procedure. One could for example use image modification software to alter artworks from the very exhibit studied and create stimuli pairs. In museum studies contexts, it would in fact probably be easier to identify subject matter experts to ensure content validity. The expert panel would then be asked which stimuli of the pair is of higher aesthetic quality, and one would select items where there is a strong or unanimous agreement (Götz et al., 1979) or keep all items and score as a function of a respondent's agreement with the expert consensus (Thorndike, 1916).

## Conclusion

In over a century of research, visual aesthetic sensitivity testing has slowly advanced toward offering test material that finally presents encouraging—although fragile—signs of validity. Both psychometric research in visual aesthetic sensitivity testing and museum research could benefit from the implementation of these tests in museum contexts. For the former, we think that it could lead to clarifying the real-world implications of visual aesthetic sensitivity; for the latter, it could prove an important factor in the understanding of individual differences between museum visitors. While speculatory at this stage, the findings previously discussed could, for example, lead to hypothesize high aesthetic sensitivity individuals to be more engaged, reflective and attentive when visiting museums and viewing artworks, to demand more cognitive stimulation (with, for example, more contextual explanations), to make longer museum visits, to compare artworks more extensively, and to be more critical of exhibited artworks. We could thus anticipate visual aesthetic sensitivity tests to be useful in better understanding the traits of a museum's or an exhibition's audience—in both understanding who the typical visitor is, and in how different the visitors may be in their approach to art—and it thus may be useful in tailoring the museum experience to better anticipate and respond to the visitors' characteristics.

## Author Contributions

NM proposed and drafted the paper. NM and FZ participated in its conceptualization and FZ made important modifications to enhance the quality of the paper.

### Conflict of Interest

The authors declare that the research was conducted in the absence of any commercial or financial relationships that could be construed as a potential conflict of interest.
